# Engineering a PAM-flexible SpdCas9 variant as a universal gene repressor

**DOI:** 10.1038/s41467-021-27290-9

**Published:** 2021-11-25

**Authors:** Jian Wang, Yuxi Teng, Ruihua Zhang, Yifei Wu, Lei Lou, Yusong Zou, Michelle Li, Zhong-Ru Xie, Yajun Yan

**Affiliations:** 1 School of Chemical, Materials and Biomedical Engineering, College of Engineering, University of Georgia, Athens, GA 30602 USA; 2 School of Electrical and Computer Engineering, College of Engineering, University of Georgia, Athens, GA 30602 USA; 3 North Oconee High School, Bogart, GA 30622 USA

**Keywords:** CRISPR-Cas systems, Synthetic biology

## Abstract

The RNA-guided CRISPR-associated Cas9 proteins have been widely applied in programmable genome recombination, base editing or gene regulation in both prokaryotes and eukaryotes. SpCas9 from *Streptococcus pyogenes* is the most extensively engineered Cas9 with robust and manifold functionalities. However, one inherent limitation of SpCas9 is its stringent 5′-NGG-3′ PAM requirement that significantly restricts its DNA target range. Here, to repurpose SpCas9 as a universal gene repressor, we generate and screen variants of the deactivated SpCas9 (SpdCas9) with relaxed 5′-CAT-3′ PAM compatibility that can bind to the start codon ATG of almost any gene. Stepwise structure-guided mutations of the PAM-interacting residues and auxiliary PAM-proximal residues of the SpdNG (5′-NG-3′ PAM) create a PAM-flexible variant SpdNG-LWQT that preferentially accommodates 5′-NRN-3′ PAMs. SpdNG-LWQT is demonstrated to be effective in gene repression with the advantage of customizable sgRNA design in both *Escherichia coli* and *Saccharomyces cerevisiae*. This work validates the feasibility of purposeful PAM expansion of Cas9 towards signature PAMs and establishes a universal SpdCas9-based gene repressor.

## Abstract (web summary)

SpCas9 has a NGG PAM requirement that limits its DNA target range. Here the authors present a PAM-expanded SpdCas9 variant SpdNG-LWQT to target NRN PAMs.

## Introduction

The bacterial type II clustered, regularly interspaced, short palindromic repeats (CRISPR/Cas9) systems have been extensively harnessed for programmable genome editing in both prokaryotic and eukaryotic cells via RNA-guided cleaving or nicking the gene target of interest^1–5^. Engineering and repurposing the nuclease-deficient Cas9 (dCas9) has derived various applications, including base editing, DNA transposition, gene interference (CRISPRi), and activation (CRISPRa)^6–12^. Recruitment of (d)Cas9 to target DNA needs two specificity checkpoints: either a dual CRISPR RNA (crRNA)-trans-activating CRISPR RNA (tracrRNA) guide or a chimeric single-molecule guide RNA (sgRNA) that specifically pairs with the target DNA strand, and a protospacer adjacent motif (PAM) immediately downstream of the protospacer on the complementary strand^13^. The sgRNA consists of a Cas9-binding RNA structure and a target-specific complementary region, rendering it easily reprogrammable to target virtually any genomic site. However, the stringent PAM requirement is a major constraint that limits the targeting scope of (d)Cas9 and thus its wide applications, especially when precise positioning is required.

The most direct solution to ease the PAM restriction would be to engineer Cas9 variants with altered or broader PAM specificities. Although mining Cas9 orthologs from a multitude of microbial resources could potentially identify Cas9s with alternate or minimal PAMs, protein characterization as well as application validation in various genetic contexts is still laborious^14–17^. Instead, engineering well-characterized Cas9s to recognize target or expanded PAMs would be more straightforward and purposeful^18,19^. Among known Cas9s, *Streptococcus pyogenes* Cas9 (SpCas9) has been most extensively engineered due to its wide and robust in vivo applications. Structure–activity relationship investigations on the SpCas9–sgRNA–DNA complex have revealed its PAM recognition mechanism, laying the structural basis for rewiring the PAM preferences via protein engineering^20–23^. SpCas9 recognizes the 5′-NGG-3′ PAM by a pair of arginine residues (R1333/R1335) within the PAM-interacting (PI) domain inserted into the major groove of the PAM DNA duplex^20^. In order to modify the PAM specificity, earlier efforts utilized directed evolution to generate the VQR, EQR, and VRER variants that recognize altered 5′-NGA-3′, 5′-NGAG-3′, and 5′-NGCG-3′ PAMs, respectively^24–26^. Since then, intensive efforts have been focused on creating SpCas9 variants with relaxed PAM specificities, ranging from xCas9 and SpCas9-NG targeting 5′-NG-3′ PAM to SpCas9-NRRH, SpCas9-NRTH, and SpCas9-NRCH that collectively recognize 5′-NRNH-3′ PAMs^19,27,28^. Very recently, a variant SpRY has been developed with almost no PAM constraint (5′-NRN-3′ > 5′-NYN-3′ PAMs)^29^. These endeavors significantly expanded the targetable space of Cas9 and demonstrated the feasibility of engineering Cas9 to accommodate noncanonical PAMs in a purpose-driven manner.

In this work, we propose to create SpdCas9 variants capable of recognizing and binding to gene start codons, which could potentially serve as universal gene repressors. In biological systems, AUG is the most commonly used start codon, whereas non-AUG start codons are rare in eukaryotic genomes, while prokaryotes permit frequent use of alternate start codons^30,31^. In *Escherichia coli*, 83% of all genes (3542/4284) start with AUG and the other 17% initiate with alternate non-AUG codons like GUG and UUG^32^. CRISPRi targeting DNA sites close to start codons could achieve a comparable inhibition efficiency as opposed to targeting the -35/-10 boxes on promoter or ribosome binding site (RBS) that are common targets for efficient gene repression^9,33^. Thus, instead of searching for the randomly distributed 5′-NGG-3′ PAM within the 5′ untranslated region, dCas9 variants recognizing the featured nucleotide triplet (ATG) could be readily repurposed as ideal gene-specific repressors with customizable sgRNA design. CRISPRi targeting start codons also minimizes any undesirable interference of upstream genes, especially considering the compact nature of prokaryotic genomes^34^.

Since RNA-directed dCas9 binding to the nontemplate DNA strand of gene coding sequence could afford effective gene repression^9,35,36^, we herein aim to mutagenize SpdCas9 to recognize the noncanonical 5′-CAT-3′ PAM for binding to the ATG start codons. By employing structure-based mutagenesis and an eGFP repression assay, we obtain one SpdCas9-NG derived variant named SpdNG-LWQT that exhibited expanded compatibility toward 5′-NRN-3′ and some 5′-NYN-3′ PAMs. We further validate its 5′-CAT-3′ PAM recognition by restoring and substantiating its nuclease activity (SpNG-LWQT) both in vivo and in vitro. Finally, with eGFP repression or mevalonate production enhancement, we demonstrate the application of SpdNG-LWQT as a universal gene repressor in both prokaryotic and eukaryotic cells. This work generates a dCas9 variant with relaxed and desired PAM specificity, which could serve as a programmable transcriptional repressor covering any gene. More broadly, the variant described here could also extend to other Cas9 or dCas9-based applications.

## Results

### Generation of dCas9 variants targeting 5′-CAT-3′ PAM

**Fig. 1:**
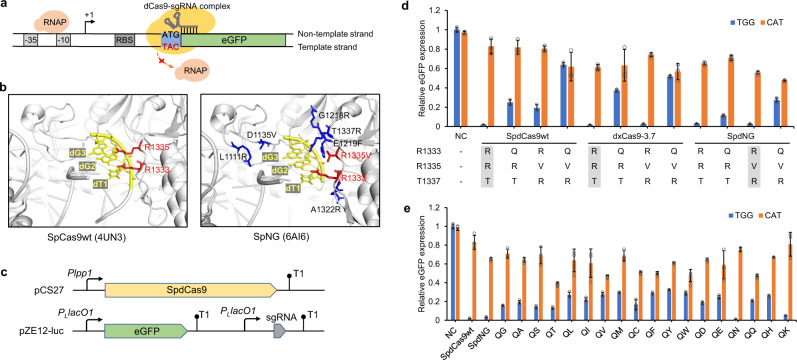
Generation of dCas9 variants targeting 5′-CAT-3′ protospacer adjacent motif (PAM) sequence. **a** Inhibition of transcription elongation from RNA polymerase (RNAP) when SpdCas9 variant was targeted to the start codon ATG of target genes. SpdCas9 variant recognizing 5′-CAT-3′ PAM sequence could bind at the start codon ATG, with sgRNA spacer complementary to the ATG adjacent sequence (20 bp) on the nontemplate DNA strand. The -35 and -10 boxes, and ribosome binding site (RBS), are shown in gray. +1, transcription initiation site. **b** PAM-interacting (red) or proximal residues (blue) in SpCas9wt (PDB ID: 4UN3) and SpNG (PDB ID: 6AI6). PAM sequence is shown in yellow. **c** The dual-plasmid eGFP repression system for SpdCas9 variant characterization: pCS27 containing *Plpp1*-controlled SpdCas9wt or variants and pZE12-luc containing *P*_*L*_*lacO1*-controlled eGFP and sgegfp-TGG or sgegfp-CAT. **d** Impact of mutating PAM interaction residues (R1333/R(V)1335/T(R)1337) in SpdCas9wt, dxCas9-3.7, and SpdNG on 5′-TGG-3′ (blue) and 5′-CAT-3′ (orange) PAM recognition. The residues in shaded boxes correspond to residues in unmutated SpdCas9wt, dxCas9-3.7, and SpdNG, respectively. **e** Impact of combinatorial mutations of R1333/V1335 in SpdNG on 5′-TGG-3′ (blue) and 5′-CAT-3′ (orange) PAM recognition. NC, *E. coli* BW25113(F′) cotransformed with the empty pCS27 plasmid and pZE-eGFP-sgegfp-TGG or pZE-eGFP-sgegfp-CAT. Data indicated the mean ± standard deviation (*n* = 3 independent biological replicates). Source data are provided as a Source Data file.

SpdCas9 variants capable of recognizing the 5′-CAT-3′ PAM could sterically block RNA polymerase (RNAP) from transcription elongation from the ATG start codon, thereby rendering gene repression (Fig. 1a). To alter its PAM specificity from 5′-NGG-3′ to 5′-CAT-3′, we started with mutating essential PI residues. In wild-type SpdCas9 (SpdCas9wt), the R1333/R1335/T1337 are close to the PAM, with side chains of R1333 and R1335 specifically interacting with the second and third guanine (G) of the cognate 5′-NGG-3′ PAM via bidentate hydrogen bonds (Fig. 1b)^25,26^. Previous studies have elucidated that R1335Q-containing SpdCas9 variants (VQR or EQR, D1135V(E)/R1335Q/T1337R) showed modified specificity to 5′-NGA-3′ PAM through the bidentate hydrogen bonding between the side chain of glutamine and adenine (A)^24,26^. This implied that R1333Q-containing SpdCas9 mutants could be potentially rewired to 5′-CAT-3′ PAM.

To test our hypothesis, we introduced R1333Q into both SpdCas9wt and the well-developed SpdCas9 variants with relaxed 5′-NG-3′ PAM specificities, including dxCas9-3.7 (D10A/E480K/E543D/E1219V/A262T/S409I/M694I/H840A) and SpdCas9-NG (SpdNG, D10A/H840A/R1335V/L1111R/D1135V/G1218R/E1219F/A1322R/T1337R) (Fig. 1b)^19,27^. For PAM determination, we employed an eGFP repression assay consisting of two plasmids, the pZE-eGFP-sgegfp harboring eGFP and sgRNA targeting eGFP, respectively, under a *P*_*L*_*lacO1* promoter, and the pCS-*Plpp1*-dCas9 harboring dCas9 under a constitutive *Plpp1* promoter^37^ (Fig. 1c). When targeting 5′-TGG-3′ PAM close to the start codon, SpdCas9wt, dxCas9-3.7, and SpdNG could efficiently repress eGFP by more than 95%, while SpdNG repressed eGFP by 44.3% with sgegfp targeting 5′-CAT-3′ PAM at the start codon (Fig. 1d). When introducing the R1333Q mutation, SpdNG R1333Q showed the best performance, retaining 72.6% repression toward 5′-TGG-3′ while achieving 52.3% repression toward 5′-CAT-3′ PAM (Fig. 1d). However, in the context of either SpdCas9wt or dxCas9-3.7, introducing R1333Q or R1333Q/R1335V/T1337R could not reach similar eGFP repression when targeting 5′-TGG-3′ or 5′-CAT-3′ PAM. With relaxed PAM recognition, SpdNG R1333Q (hereafter named as SpdNG-Q) was therefore chosen as a starting molecular scaffold for enhancing 5′-CAT-3′ recognition.

Since V1335 is critical in relaxing SpdNG PAM specificity, we then conducted scanning mutagenesis at V1335 of SpdNG-Q and tested its impact on 5′-CAT-3′ recognition. Among all substitutions, V1335T (mutant SpdNG-QT) increased compatibility toward both 5′-TGG-3′ and 5′-CAT-3′ PAMs, exhibiting 87.1% and 60% eGFP repression on TGG and CAT PAM targets, respectively (Fig. 1e). Unexpectedly, SpdNG-QN and -QK, which harbor R1333Q/V1335N or R1333Q/V1335K, showed almost uncompromised recognition on 5′-TGG-3′ PAM as SpdCas9wt.

### Structure-guided improvement of the activity of SpdNG-QT

**Fig. 2:**
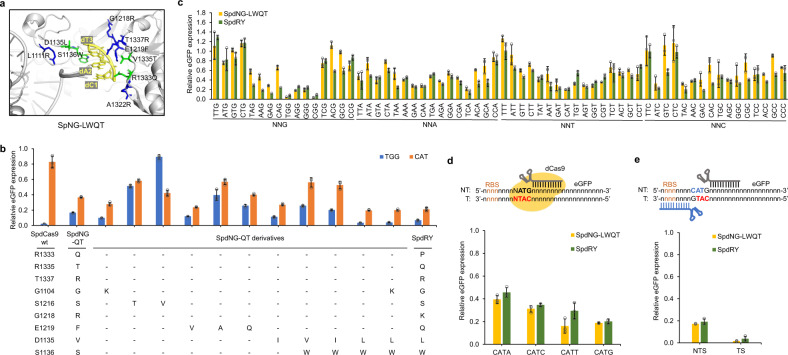
Improving the activity of SpdNG-QT toward 5′-CAT-3′ PAM by mutating PAM-proximal residues. **a** PAM-proximal residues involved in PAM recognition. Modeled structure of SpNG-LWQT (D1135L/S1136W/R1333Q/V1335T, residues shown in green) with 5′-CAT-3′ PAM based on SpNG (PDB ID: 6AI6). **b** Impact of mutating PAM-proximal residues on 5′-TGG-3′ (blue) and 5′-CAT-3′ (orange) PAM recognition. **c** PAM profile comparison between SpdNG-LWQT (gold) and SpdRY (green) against all NNN PAM libraries. **d** The influence of the fourth-position base on 5′-CAT-3′ PAM recognition of SpdNG-LWQT (gold) and SpdRY (green). **e** The repression effects of SpNG-LWQT (gold) and SpdRY (green) when targeting 5′-CAT-3′ PAM on both nontemplate and template strand. NTS nontemplate strand, TS template strand, RBS ribosome binding site. Data indicated the mean ± standard deviation (*n* = 3 independent biological replicates). Source data are provided as a Source Data file.

To further improve the activity of SpdNG-QT, we attempted to promote the stacking interaction between Cas9 and PAM by mutating the key residues around the PAM-Cas9 duplex, including G1104, D1135, S1136, S1216, and E1219 (Fig. 2a). We hypothesized that mutating these PAM adjacent residues could afford expanded PAM tolerance. In SpCas9wt, E1219 forms a salt bridge with R1335, stabilizing its PAM specificity to 5′-NGG-3′^20^. E1219 mutations have been frequently observed in evolved SpCas9 variants and demonstrated to be critical in relaxing PAM stringency^19,27,38,39^. Thus, we first focused on investigating the impact of the E1219 mutation on PAM recognition. Since SpdNG-QT harbors an E1219F mutation, we replaced F1219 with smaller residues including A, Q and V. The eGFP repression assay showed that only F1219V could further improve its activity toward 5′-CAT-3′ PAM, achieving 77.3% repression (Fig. 2b).

Residues G1104, D1135, S1136, and S1216 located at the sugar-phosphate backbone side of the PAM in SpCas9wt^20,29^ (Fig. 2a). G1104K was previously observed to increase nonspecific DNA contacts and facilitate altered PAM tolerance^29^, whose introduction into SpdNG-QT increased eGFP repression to 73.4% when targeting 5′-CAT-3′ PAM (Fig. 2b). To push the PAM DNA toward R1333Q/R1335T, we first mutated S1216 to T or V. However, both of them significantly impaired the recognition toward either 5′-TGG-3′ or 5′-CAT-3′ PAM (Fig. 2b). We then introduced single or combinatorial mutations of V1135 and S1136 into SpdNG-QT or its G1104K mutant. The combinatorial mutant SpdNG-QT V1135L/S1136W (named SpdNG-LWQT) afforded the highest activity toward both 5′-TGG-3′ and 5′-CAT-3′ PAMs, exhibiting 96.5% and 80.4% eGFP repression, respectively (Fig. 2b). In addition, SpdNG-LWQT slightly outperformed the previously engineered SpdRY mutant on both PAMs (Supplementary Fig. 1), which is the most PAM-flexible SpdCas9 variant obtained so far^29^. To thoroughly profile the PAM range of SpdNG-LWQT, we incorporated nucleotide triplets adjacent to the start codon of eGFP, generating an NNN PAM containing plasmid library, pZE-NNN-eGFP-sgegfp. The repression profile indicated that SpdNG-LWQT showed a similar PAM profile as SpdRY (5′-NRN-3′ > 5′-NYN-3′) but favored 5′-NGG-3′ and most 5′-NRT-3′ PAMs tested (Fig. 2c).

The fourth-base G in the PAM has been reported critical or biased for T1337R-containing SpdCas9 mutants including SpdNG and SpdRY, due to the direct contact of T1337R with the fourth G^25,26,29,40^. To investigate the impact of the fourth-position nucleotide on 5′-CAT-3′ PAM recognition by SpdNG-LWQT, we created 5′-CATN-3′ PAMs at the ATG start codon of eGFP (Fig. 2d). The eGFP repression assay indicated that SpdNG-LWQT could accommodate all 5′-CATN-3′ PAMs with 60.5-83.9% repression efficiencies (CATT = CATG > CATC > CATA), which were also higher than those with SpdRY (Fig. 2d). Next, to examine the effects of targeting DNA strand on gene repression, we designed sgegfp targeting the template DNA strand with a 5′-CAT-3′ PAM on the complementary strand (Fig. 2e). Interestingly, SpdNG-LWQT targeting template DNA strand afforded even higher repression efficiency (98.2%), probably ascribed to the binding of dCas9–sgRNA complex to the RBS^35^ (Fig. 2e). Very recently, a SpCas9-VRKG variant (D1135V/S1136R/D1332K/R1333G) recognizing 5′-RNG-3′ PAMs was created^41^. When repurposing the SpdCas9-VRKG for gene repression at the 5′-ATG-3′ PAM with sgegfp targeting the template strand, it showed much lower repression efficiency than SpdNG-LWQT targeting 5′-CAT-3′ PAM on either DNA strand (Supplementary Fig. 2). These results together demonstrated that SpdNG-LWQT could enable efficient gene repression at ATG start codons.

### Verification of engineered Cas9 variants via DNA cleavage assay

**Fig. 3:**
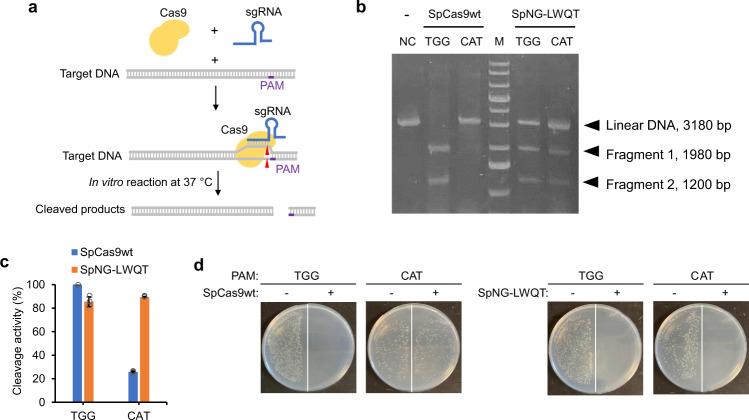
Restoring the nuclease activity SpdNG-LWQT toward 5′-TGG-3′ and 5′-CAT-3′ PAMs. **a** In vitro DNA cleavage assay for Cas9 activity. Purified his_6_-tagged Cas9 proteins were incubated with in vitro transcribed sgRNA and linearized DNA targets, and the reaction was performed at 37 °C. **b** DNA cleavage by SpCas9wt and SpNG-LWQT with sgegfp targeting 5′-TGG-3′ or 5′-CAT-3′ PAM. DNA target with SpCas9wt but without any sgRNA was served as the negative control. Representative image from two independent repeats. **c** In vivo plasmid DNA cleavage assay with SpdCas9wt (blue) and SpNG-LWQT (orange). Plasmid pCS-*Plpp1*-SpCas9wt or pCS-*Plpp1*-SpNG-LWQT was transferred into *E. coli* BW25113(F′) harboring pZE-eGFP-sgegfp containing sgegfp targeting 5′-TGG-3′ or 5′-CAT-3′ PAM targets on eGFP. Data indicated the mean ± standard deviation (*n* = 3 independent biological replicates). **d** In vivo chromosomal DNA cleavage assay. Plasmid pCS-*Plpp1*-SpCas9wt or pCS-*Plpp1*-SpNG-LWQT was transferred into *E. coli* BW25113(F′)::eGFP harboring pZE-sgegfp containing sgegfp targeting 5′-TGG-3′ or 5′-CAT-3′ PAM targets on chromosomally integrated eGFP. Cells were plated on Luria-Bertani (LB) plates containing 0.5 mM IPTG and appropriate antibiotics. Negative controls (−) were transformed with empty pCS27 plasmid. Representative image from two independent repeats. Source data are provided as a Source Data file.

To substantiate the PAM recognition of the engineered SpdNG-LWQT variant, we restored its nuclease activity by introducing A10D and A840H mutations. The resultant nuclease-active SpNG-LWQT was expressed in *E. coli* BL21 Star(DE3) and purified to homogeneity for in vitro DNA cleavage assay (Fig. 3a). As a control, SpCas9wt could cleave the linearized pZE-eGFP-sgegfp plasmid at 5′-TGG-3′ PAM, but could barely cleave at 5′-CAT-3′ PAM. Instead, SpNG-LWQT could cleave the plasmid DNA at both 5′-TGG-3′ and 5′-CAT-3′ PAMs (Fig. 3b).

To further validate its PAM recognition activity in vivo, we tested the DNA cleavage activity of SpNG-LWQT using a cell survival assay. For plasmid DNA cleavage, pCS-*Plpp1*-SpNG-LWQT was transferred into *E. coli* BW25113(F′) harboring pZE-eGFP-sgegfp. When sgegfp transcription was induced by IPTG, cleavage of pZE-eGFP-sgegfp would deplete ampicillin resistance. SpNG-LWQT afforded approximately 90% plasmid cleavage against both 5′-TGG-3′ and 5′-CAT-3′ PAMs (Fig. 3c). As a control, SpCas9wt achieved 100% plasmid cleavage with 5′-TGG-3′ but only 25.8% with 5′-CAT-3′ PAM. Considering the wide application of Cas9 for genome editing, we then tested the cleavage activity of SpNG-LWQT on genomic DNA. An engineered strain *E. coli* BW25113(F′)::eGFP was created by integrating the *P*_*L*_*lacO1-*eGFP cassette into the chromosome of *E. coli* BW25113(F′) between the *nupG* and *speC* loci^33,42^, in which eGFP cleavage would result in cell death. When transformed with pZE-sgegfp targeting 5′-TGG-3′ PAM, both SpCas9wt and SpNG-LWQT could deprive the cell of growth, while only the latter is lethal when transforming pZE-sgegfp targeting 5′-CAT-3′ PAM (100% cleavage) (Fig. 3d). The DNA cleavage results demonstrated that the engineered SpNG-LWQT showed expanded compatibility to 5′-CAT-3′ PAM.

### PAM interaction analysis via molecular dynamics (MD) simulations

**Fig. 4:**
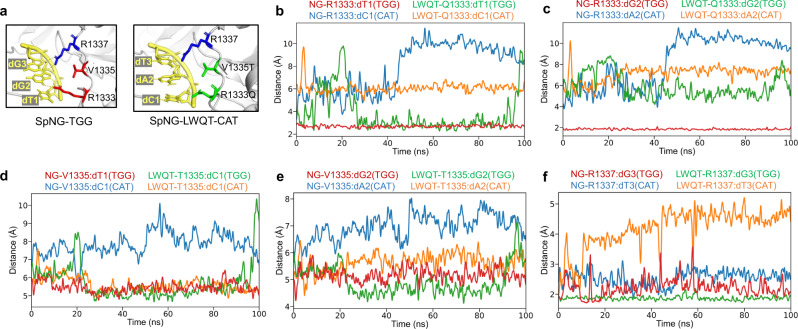
The minimum distances between amino acids and nucleotides. **a** Overview of PAM interactions between SpNG (PDB ID: 6AI6) or SpNG-LWQT and 5′-TGG-3′ or 5′-CAT-3′ PAMs. **b** The minimum distance between R(Q)1333 and dT(C)1. **c** The minimum distance between R(Q)1333 and dG(A)2. **d** The minimum distance between V(T)1335 and dT(C)1. **e** The minimum distance between V(T)1335 and dG(A)2. **f** The minimum distance between R1337 and dG(T)3. Red line: SpNG-TGG; blue line, SpNG-CAT; green line, SpNG-LWQT-TGG; yellow line, SpNG-LWQT-CAT.

To support the evidence that mutated Cas9 can recognize 5′-CAT-3′ PAM, we conducted MD simulations to predict molecular interactions between the PI domain and the PAMs. Four independent simulations were performed by using SpNG (PDB ID: 6AI6) or its mutant SpNG-LWQT binding to 5′-TGG-3′ or 5′-CAT-3′ PAM (i.e., SpNG-TGG, SpNG-CAT, SpNG-LWQT-TGG, and SpNG-LWQT-CAT) (Fig. 4a). Based on trajectory files, we calculated the minimum distances between PAMs and the residues on the PI domain. As a result, we found that the minimum distance between R1333 and dC1 (or dA2) on SpNG-CAT is longer than 6.0 Å and also longer than other structures after 45 ns, suggesting that R1333 can scarcely interact with 5′-CAT-3′ PAM (Fig. 4b, c). A similar scenario occurred between V1335 and dC1 (or dA2) (Fig. 4d, e). Notably, after we mutated four amino acids on the PI domain (V1135L/S1136W/R1333Q/V1335T), we found that the minimum distance between Q1333 and dC1 (or dA2) is shorter than that of R1333 and dC1 (or dA2) on SpNG-CAT after 45 ns (Fig. 4b, c). For T1335 and dC1 (or dA2), the minimum distance is shorter than V1335 and dC1 (or dA2) after about 5 ns (Fig. 4d, e). These results demonstrated that the mutated PI domain in SpNG-LWQT may have slightly stronger interaction with PAM than that on SpNG. In addition, the mutated SpNG-LWQT also interacts with 5′-TGG-3′ PAM. Except that the distance between R1333 and dG2 is significantly shorter than the distance between Q1333 and dA2 (Fig. 4c), we found that the minimum distances between Q1333 (or T1335) and dT1 (or dG2) on SpNG-LWQT-TGG are similar or even shorter compared to the distance between R1333 (or V1335) and dT1 (or dG2) (Fig. 4b, d, e). For R1337 and dG3, the minimum distance on SpNG-LWQT-TGG is shorter than that on SpNG-TGG (Fig. 4f). These results showed that SpNG-LWQT can interact with not only 5′-TGG-3′ PAM but also 5′-CAT-3′ PAM, suggesting that the ability of Cas9 to interact with diverse PAM sequences is improved.

### Engineered SpdNG-LWQT as a universal gene repressor in *E. coli* and yeast

SpdCas9 has been demonstrated to be a robust gene regulation tool guided by programmable sgRNAs. However, the wide use of SpdCas9 is limited by two major constraints: (1) the stringent PAM requirement that hinders targeting range, and (2) the strong gene repression that affects cell fitness when targeting essential genes^36,43,44^. With flexible PAM requirements and relaxed repression effects, SpdNG-LWQT would serve as a more suitable gene repressor rendering customizable sgRNA design and tunability.

**Fig. 5:**
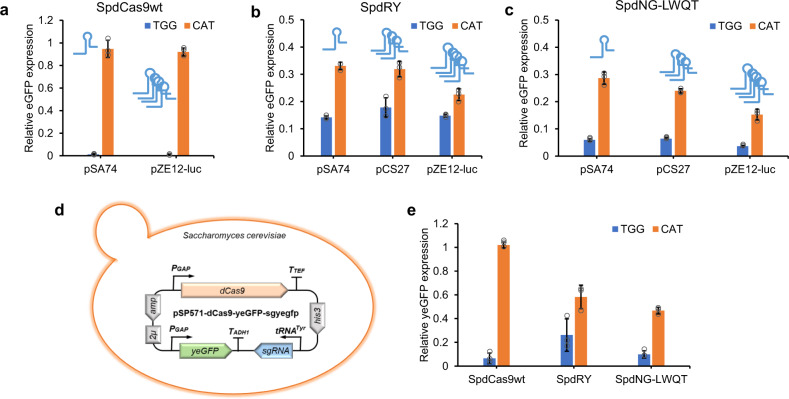
Transcriptional repression of eGFP in *E. coli* and *S. cerevisiae*. Repression of chromosomal eGFP by SpdCas9wt (**a**), SpdRY (**b**) and SpdNG-LWQT (**c**) targeting both 5′-TGG-3′ (blue) and 5′-CAT-3′ (orange) PAMs in *E. coli* BW25113(F′)::eGFP. The dCas9s were expressed on pCS27, and sgRNAs were expressed on low-copy (pSA74), medium-copy (pCS27), or high-copy (pZE12-luc) plasmids. **d** Construction of pSP571-dCas9-yeGFP-sgyegfp plasmid for gene repression assay in *S. cerevisiae*. The dCas9s and the yeast eGFP (yeGFP) were driven by the *P*_*GAP*_ promoter, and sgRNAs were driven by the *tRNA*^*Tyr*^ promoter. **e** Comparison of yeGFP repression by SpdCas9wt, SpdRY and SpNG-LWQT targeting both 5′-TGG-3′ (blue) and 5′-CAT-3′ (orange) PAMs in *S. cerevisiae*. Data indicated the mean ± standard deviation (*n* = 3 independent biological replicates). Source data are provided as a Source Data file.

To demonstrate the applicability of SpdNG-LWQT as a gene repressor, we first tested its repression efficacy on chromosomally integrated eGFP in *E. coli*. For simplicity, dCas9s were placed on the medium-copy plasmid pCS27, and sgRNAs were, respectively, incorporated into the low-copy (pSA74), medium-copy (pCS27), and high-copy plasmid (pZE12-luc). When transferred into the host strain *E. coli* BW25113(F′)::eGFP, SpdCas9wt almost completely silenced eGFP expression with sgRNA targeting 5′-TGG-3′ PAM (>99% repression), but could hardly repress eGFP when targeting 5′-CAT-3′ PAM, regardless of the sgRNA expression level (Fig. 5a). While recognizing both PAMs, SpdNG-LWQT outperformed SpdRY against either 5′-TGG-3′ or 5′-CAT-3′ PAM (Fig. 5b, c). With 5′-TGG-3′ PAM, the eGFP repression efficiency of SpdNG-LWQT could reach more than 94% with sgRNA on different plasmids (Fig. 5c). Particularly, SpdNG-LWQT exhibited tunability of repression efficiency when targeting 5′-CAT-3′ PAM, ranging from 71.3% to 84.7% when gradually increasing sgRNA copies (Fig. 5b, c). These results corroborated that SpdNG-LWQT could enrich the dCas9 toolboxes as a programmable and tunable gene repressor.

To further evaluate the functionality of SpdNG-LWQT in eukaryotic cells, we constructed a single-plasmid system for gene repression assay in *Saccharomyces cerevisiae*. The constructed plasmid, pSP571-dCas9-yeGFP-sgyegfp (2μ ori, His3 selection marker), harbors the yeast codon-optimized SpdNG-LWQT and a yeast-enhanced eGFP (yeGFP), respectively, driven by *P*_*GAP*_ promoters, and the sgRNA under control of *tRNA*^*Tyr*^ promoter^45,46^ (Fig. 5d). Yeast codon-optimized SpdCas9wt and SpdRY were applied as controls. The sgRNA was designed to target either 5′-TGG-3′ PAM near the start codon or 5′-CAT-3′ PAM at the start codon of yeGFP. When transferred into the host *S. cerevisiae* BY4741, SpdCas9wt achieved 93.5% yeGFP repression against 5′-TGG-3′ PAM but no repression against 5′-CAT-3′ PAM, while SpdRY exerted 73.8% and 41.7% yeGFP repression against these two PAMs (Fig. 5e). SpdNG-LWQT afforded even higher repression efficacies than SpdRY, reaching 90.1% and 53.3% against both PAMs, respectively (Fig. 5e). These results together indicated that SpdNG-LWQT could function as a universal gene repressor in both prokaryotic and eukaryotic cells.

### Mevalonate production enhancement via SpdNG-LWQT mediated CRISPRi

**Fig. 6:**
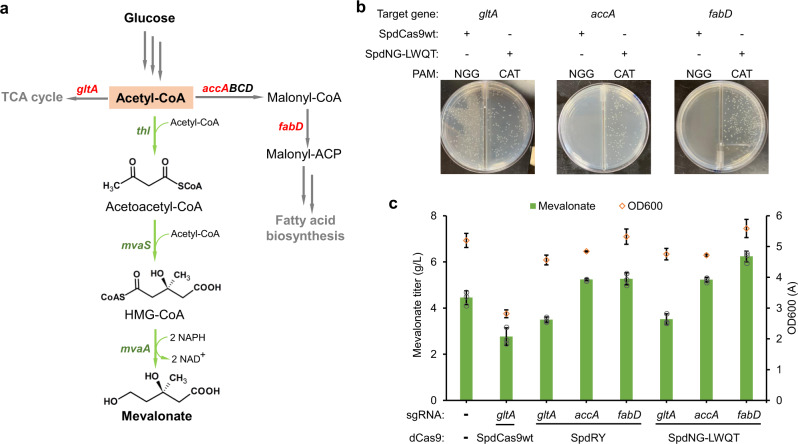
Production enhancement of mevalonate via SpdNG-LWQT mediated CRISPR interference (CRISPRi). **a** The metabolic pathway of mevalonate from glucose. The pathway consists of three enzymes: thiolase (*thl*) from *C. difficile*, 3-hydroxy-3-methyl-glutaryl-coenzyme A (HMG-CoA) synthase (*mvaS*) from *L. casei*, and HMG-CoA reductase (*mvaA*) from *R. pomeroyi*. Three competing essential genes (shown in red) from *E. coli* are: *gltA* encoding citrate synthase, *accA* encoding the subunit α of acetyl-CoA carboxyltransferase, and *fabD* encoding malonyl-CoA-acyl carrier protein transacylase. **b** The influence of CRISPRi of *gltA*, *accA*, or *fabD* on cell fitness of *E. coli* BW25113(F′)::MVA. The dCas9s were expressed on pCS27, and sgRNAs were expressed on pZE12-luc. All sgRNAs were targeting coding sequence on nontemplate DNA strand. SpdCas9wt was coexpressed with sgRNAs targeting 5′-NGG-3′ PAM while SpdRY and SpdNG-LWQT were coexpressed with sgRNAs targeting 5′-CAT-3′ PAM at the ATG start codons. Cells were plated on Luria-Bertani (LB) plates containing 0.5 mM IPTG and appropriate antibiotics. Representative image from two independent repeats. **c** The effects of SpdCas9wt or its variants mediated CRISPRi on mevalonate production in *E. coli* host BW25113(F′)::MVA. The host strain with empty pCS27 and pZE12-luc was applied as a negative control (NC). All shake flasks were performed in M9 minimal medium containing 20 g/L glucose and 5 g/L yeast extract, and samples were taken at 48 h. Data indicated the mean ± standard deviation (*n* = 3 independent biological replicates). Source data are provided as a Source Data file.

To showcase the application of SpdNG-LWQT mediated CRISPRi, we implemented it to mevalonate production enhancement. The mevalonate pathway starts with the condensation of three acetyl-CoA into 3-hydroxy-3-methyl-glutaryl-coenzyme A (HMG-CoA) via thiolase (Thl) and HMG-CoA synthase (MvaS), followed by reduction of HMG-CoA to mevalonate via HMG-CoA reductase (MvaA) with two NAD(P)H inputs (Fig. 6a). To construct a mevalonate producer, we assembled a synthetic pathway consisting of *thl* from *Clostridium difficile*, *mvaS* from *Lactobacillus casei*^47^, and an NADH-dependent *mvaA* from *Ruegeria pomeroyi*^48^, and incorporated it into the *E. coli* BW25113(F′) chromosome at *aslB* locus (yielding *E. coli* BW25113(F′)::MVA). To increase the acetyl-CoA supply for the mevalonate pathway, we chose to knockdown three competing essential genes, including the citrate synthase gene (*gltA*) for TCA cycle, the acetyl-CoA carboxyltransferase subunit α gene (*accA*), and the malonyl-CoA-acyl carrier protein transacylase gene (*fabD*) for fatty acid biosynthesis initiation (Fig. 6a). With SpdCas9wt, expressing sgRNA targeting either *accA* or *fabD* at 5′-NGG-3′ PAM near the start codon was lethal to *E. coli* BW25113(F′)::MVA, while targeting *gltA* decreased cell density by 45.9% and mevalonate titer by 38.0% in 48 h of shake flask cultivation (Fig. 6b, c). In contrast, both SpdRY and SpdNG-LWQT exhibited less or no inhibition on cell growth with sgRNAs targeting the start codon (5′-CAT-3′ PAM) of all three essential genes (Fig. 6b, c). SpdRY and SpdNG-LWQT increased mevalonate titers when repressing either *accA* or *fabD*. Particularly, SpdNG-LWQT targeting *fabD* afforded the highest mevalonate titer of 6.24 g/L, accounting for a 40.1% increase compared with the control strain without CRISPRi (4.45 g/L) (Fig. 6b, c). These results demonstrated the applicability of SpdNG-LWQT mediated CRISPRi, especially when targeting essential genes.

## Discussion

The RNA-directed Cas9 nucleases have been adapted for multiple applications with programmability, covering genome recombination, base editing, DNA transposition and gene regulation, etc. The stringent PAM requirement is crucial for Cas9 DNA specificity, which however, also restricts the targeting scope and thus the wide applications of the Cas9 toolkits. Although the most widely used SpCas9 has been overwhelmingly engineered to rewire its PAM specificity from the cognate 5′-NGG-3′ to altered or expanded PAMs, most of them are G-containing PAMs^19,25,27^. Very recently, SpCas9 variants recognizing non-G PAMs, including the near-PAMless variant SpRY (5′-NRN-3′ > 5′-NYN-3′ PAMs), have been created via directed evolution or systematic mutagenesis^28,29^. In this research, to generate a gene-specific repressor that blocks transcription elongation at the start codon ATG, we created a PAM-expanded SpdCas9 variant SpdNG-LWQT with compatibility to the counterpart non-G 5′-CAT-3′ PAM. This endeavor would enrich the SpCas9 toolboxes with expanded targeting scopes and offer a unique variant suitable as a universal gene repressor.

The ability of evolved SpCas9 variants xCas9-3.7 and SpNG to recognize expanded 5′-NG-3′ PAMs provides potential molecular scaffolds for PAM rewiring. To facilitate PAM profiling, we utilized an eGFP repression assay with the nuclease-inactive SpdCas9 or its variants, where PAM recognition relies on DNA target binding. Considering that Gln could form hydrogen bonds with adenine, we introduced R1333Q to increase potential contact with the second-position adenine of 5′-CAT-3′ PAM. Our initial efforts identified that R1333Q-harboring SpdNG (SpdNG-Q) showed partial recognition on 5′-CAT-3′ and retained recognition on 5′-TGG-3′. To further improve its recognition, scanning mutagenesis of the neighboring V1335 (R1335 in SpCas9wt) yielded SpdNG-QT (R1333Q/V1335T) showing improved recognition on both 5′-CAT-3′ and 5′-TGG-3′ PAMs. Based on that, we then introduced mutations on PAM-proximal residues that could potentially influence PAM recognition, either by increasing DNA contacts or by pushing the sugar-phosphate backbone toward PI residues. The final mutant, SpdNG-LWQT (V1135L/S1136W/R1333Q/V1335T), afforded the highest eGFP repression toward both 5′-TGG-3′ and 5′-CAT-3′ PAMs, which even outperformed the near-PAMless SpdRY variant (Fig. 2b and Supplementary Fig. 1). Further examination of PAM profile confirmed that SpdNG-LWQT exhibited a similar PAM range as SpdRY, and showed higher activities toward 5′-NGG-3′ and most 5′-NRT-3′ PAMs (Fig. 2c). The PAM recognition of SpdNG-LWQT was corroborated by restoring its nuclease activity toward the eGFP target both in vitro and in *E. coli* cells (Fig. 3). The following MD simulations predicted that SpNG-LWQT retained stable hydrogen bonding between R1337 and the third G of 5′-TGG-3′, and showed shorter minimum distances between PI residues and 5′-CAT-3′ than SpNG, which potentially explains the PAM tolerance of SpdNG-LWQT toward both 5′-TGG-3′ and 5′-CAT-3′.

By targeting to start codons, SpdNG-LWQT could serve as a promising dCas9-based universal gene repressor with programmability and tunability. SpdCas9 has been demonstrated with high efficacy and wide applications in gene repression at the transcriptional level^11,49,50^. However, the near-knockout repression of target genes, especially the essential ones, may cause cell fitness defects and limitations for dCas9-based genetic circuits^36,43,51^. SpdNG-LWQT could instead afford relaxed gene repression at the start codons, and achieve tunability simply by modulating the sgRNA copies (Fig. 5c). When needed, targeting 5′-NGG-3′ PAM via SpdNG-LWQT could further increase repression efficiency to more than 95%. As showcased in mevalonate pathway, SpdNG-LWQT mediated CRISPRi of *fabD* in *E. coli* achieved 40.1% increase of mevalonate titer, in stark contrast to growth defects with SpdCas9wt mediated one (Fig. 6). Noteworthy, SpdNG-LWQT outperformed SpdRY toward 5′-NGG-3′ and 5′-CAT-3′ PAMs in both *E. coli* and *S. cerevisiae*, indicating its host flexibility (Fig. 5). In addition, SpdNG-LWQT would make the sgRNA design customizable, with the spacer complementary to the nontemplate DNA strand adjacent to start codons. All of these features render SpdNG-LWQT a promising tunable gene repressor.

In conclusion, to create a universal gene repressor targeting gene start codons, we engineered an SpdCas9 variant with an expanded PAM range including a signature 5′-CAT-3′ PAM. This exemplified the viability of harnessing PAM-flexible Cas9s for purposeful PAM alteration. The resultant SpdNG-LWQT adds to the SpdCas9-based repressors with customizable sgRNA design, tunability and host flexibility, three features that could readily render implementation into dCas9-based genetic circuits or gene control systems in a broad range of organisms. More broadly, SpdNG-LWQT could complement SpdRY to eventually permit unrestrictive access of genome targets for related genetic manipulations.

## Methods

### Bacteria, plasmids, and chemicals

Bacterial strains and plasmids used in this study were listed in Supplementary Table 1. SpCas9 was amplified from the genomic DNA of *S. pyogenes* Strain SF370 (ATCC, Manassas, VA). *E. coli* XL1-Blue (Stratagene, La Jolla, CA) was used for plasmid construction and *E. coli* BL21 Star(DE3) (Invitrogen, Waltham, MA) was employed for SpCas9 expression and purification. *E. coli* BW25113(F′) was the bacterial host for in vivo eGFP repression or cleavage assays. Plasmids pZE12-luc (high-copy), pCS27 (medium-copy), pSA74 (low-copy), and pETDuet-1 were used for gene expression. *Saccharomyces cerevisiae* BY4741 (4040002, ATCC, Manassas, VA) was used as the yeast host. Yeast plasmids pSP571 (#139498), pZ_P-GAP-eGFP (#126717), and pCAS (#60847) were purchased from Addgene (Watertown, MA). Phusion DNA polymerase, restriction endonucleases, Quick Ligation kit, T7 RNAP, DNase I, and the Monarch^®^ RNA Cleanup kit were purchased from New England Biolabs (Ipswich, MA). Yeast extract peptone dextrose (YPD) broth, yeast nitrogen base (YNB) medium, yeast synthetic drop-out medium supplements, and standard chemicals were purchased from Sigma-Aldrich (St. Louis, MO) unless otherwise specified.

### Plasmids and bacterial strains construction

DNA manipulations were conducted following the standard molecular cloning protocols^52^. The *cas9* from *S. pyogenes* was amplified and inserted into pCS27 in between *Acc65*I and *Bam*HI under control of *Plpp1* promoter^37^, yielding plasmid pCS-*Plpp1*-SpCas9wt. Site-directed mutagenesis to obtain SpdCas9wt (D10A/H840A) and its derived variants were performed using the method described by Chiu et al.^53^. The reporter plasmid pZE-eGFP was constructed by inserting eGFP into pZE12-luc in between *Acc65*I and *Xba*I. The synthesized sgegfp was inserted into pCS27 under the control of the *P*_*L*_*lacO1* promoter. The *P*_*L*_*lacO1*-sgegfp cassette was then amplified from pCS-sgegfp and inserted into pZE-eGFP in between *Spe*I and *Sac*I, yielding pZE-eGFP-sgegfp. To generate an NNN PAM library containing eGFP, the NNN nucleotides were placed adjacent to the start codon ATG of eGFP during primer design and introduced into the plasmid pZE-eGFP-sgegfp. For NNN insertions that introduce stop codons (TAA, TAG, and TGA), sgRNA spacers were instead mutated to target corresponding PAMs near the start codon of eGFP. SpCas9 or its variants were cloned into pETDuet-1 under the control of the T7 promoter. The mevalonate pathway plasmid pCS-*thl-mvaS-mvaA* was constructed by amplifying and inserting *thl* from *C. difficile*, *mvaS* from *L. casei*, and *mvaA* from *R. pomeroyi* into pCS27. The *P*_*L*_*lacO1*-*thl-mvaS-mvaA* cassette was then amplified and integrated into *E. coli* BW25113(F′) via λ-Red recombination^54^, resulting in *E. coli* BW25113(F′)::MVA. To repress competing genes for mevalonate pathway, sgRNA targeting *gltA*, *accA* and *fabD* were constructed into pZE12-luc, yielding pZE-*sggltA*, pZE-*sgaccA*, and pZE-*sgfabD*, respectively. *P*_*GAP*_-controlled yeGFP cassette was amplified from pZ_P-GAP-eGFP and inserted into pSP571 using *Spe*I and *Bam*HI, yielding pSP571-yeGFP. The *tRNA*^*Tyr*^ promoter controlled sgRNA module was amplified from pCAS and inserted into pSP571-yeGFP between *Sph*I and *Sac*I, yielding pSP571-yeGFP-sgyegfp. Yeast codon-optimized SpdCas9wt under control of *P*_*GAP*_ promoter was amplified from pSP571 and inserted into pSP571-yeGFP-sgyegfp by *Hind*III and *Spe*I, generating pSP571-SpdCas9wt-yeGFP-sgyegfp. Yeast SpdCas9 variants and sgRNA spacers were mutated using the method described by Chiu et al.^53^. All plasmids or bacterial strains involved in this study were listed in Supplementary Table 1.

### Culture media and conditions

Luria-Bertani (LB) medium (10 g/L tryptone, 5 g/L yeast extract and 10 g/L sodium chloride) was used for *E. coli* cultivation and plasmid propagation. Cells were cultivated at 37 °C in a rotary shaker at 270 rpm. The M9 minimal medium (6 g/L Na_2_HPO_4_, 0.5 g/L NaCl, 3 g/L KH_2_PO_4_, 1 g/L NH_4_Cl, 1 mM MgSO_4_, 0.1 mM CaCl_2_) containing 20 g/L glucose and 5 g/L yeast extract, was used for shake flask experiments in 125-mL conical shake flasks. The antibiotics ampicillin (100 mg/L), kanamycin (50 mg/L), chloramphenicol (34 mg/L), and isopropyl β-D-1-thiogalactopyranoside (IPTG, 0.5 mM) were added into the medium when needed. *S. cerevisiae* BY4741 was cultivated in YPD broth or YNB medium with synthetic drop-out supplement (without histidine) at 30 °C in a rotary shaker at 270 rpm.

### The eGFP repression assay

To determine the repression efficiency of SpCas9 or its variants, reporter plasmids (pZE-eGFP-sgegfp or pZE-NNN-eGFP-sgegfp) were cotransferred with the pCS27 plasmids carrying SpdCas9wt or its variants into *E. coli* BW25113(F′) cells. Reporter plasmids with the empty pCS27 were cotransferred into *E. coli* BW25113(F′) cells as controls. Single colonies were picked and inoculated in 3 mL LB tubes with appropriate antibiotics and IPTG. For yeast eGFP repression assay, pSP571-dCas9-yeGFP-sgyegfp or its derivative plasmids were transformed into *S. cerevisiae* BY4741. pSP571-yeGFP-sgyegfp was used as the control. Single yeast colonies were picked and inoculated in 3 mL histidine drop-out YNB medium. After 24 h, 20 μL culture was sampled and diluted with 180 μL distilled H_2_O in a black 96-well plate. Cell optical density at 600 nm (OD_600_) was measured and eGFP fluorescence was detected using an excitation filter of 485/20 nm and an emission filter of 528/20 nm with a Synergy microplate reader (BioTek, Winooski, VT). The relative eGFP expression was the ratio of the normalized eGFP fluorescence per OD600 (RFU/OD_600_) of cells with dCas9s to those without dCas9s.

### Shake flask experiments

For mevalonate production, *E. coli* BW25113(F′)::MVA was cotransformed with pZE12-luc derived plasmids harboring sgRNAs and pCS27 derived plasmids harboring SpdCas9wt or its variants. Empty pZE12-luc and pCS27 were used as the negative control. The shake flask experiment was conducted in a rotary shaker (New Brunswick Scientific, Edison, NJ) at 30 °C with a speed of 270 rpm. Transformants of *E. coli* BW25113(F′)::MVA were inoculated in 3 mL LB medium and grown at 37 °C for 8–10 h. The seed cultures were then transferred to 20 mL fresh M9 minimal medium containing 20 g/L glucose and 5 g/L yeast extract in 125-mL shake flasks as 2% (v/v) inoculum and grown at 30 °C for 48 h. IPTG was added with a final concentration of 0.5 mM during initial inoculation.

### Protein purification and in vitro cleavage assay

For protein purification of SpCas9 and its variants, the pETDuet-1 derived plasmids were transferred into *E. coli* BL21 Star(DE3). The transformants were inoculated in 3 mL LB tubes at a 37 °C shaker at 270 rpm. Two hundred microliters of overnight cultures were transferred into a 250-mL shaker with 50 mL fresh LB medium. When the OD_600_ reached around 0.6, 0.5 mM IPTG was added and cells were transferred to a 30 °C shaker for production induction and expression overnight. The cells were collected by centrifuging at 9391 × *g* for 10 min and then lysed using Mini Bead Beater (Biospec). Protein purification was performed using His-Spin Protein Miniprep Kit (Zymo Research, Irvine, CA) following manufacturers’ instructions. The purified protein was verified by SDS-polyacrylamide gel electrophoresis (SDS-PAGE) using 12% protein gel and the protein concentration was measured using a Pierce BCA Protein Assay Kit (Thermo Scientific, Waltham, MA) as manufacturers’ instructions.

For in vitro DNA cleavage assay, sgRNA was prepared by in vitro transcription using T7 RNAP, the products were digested with DNase I and purified with Monarch^®^ RNA Cleanup kit following manufacturers’ instructions. The sgRNA was quantified using NanoDrop 2000c spectrophotometer (Thermo Scientific, Waltham, MA). 100 nM of purified His_6_-tagged SpCas9 or its variants and 30 µM sgRNA were incubated with *Sac*I-linearized reporter plasmid pZE-eGFP-sgegfp (15 µg, 500 nM) for in vitro cleavage. The reaction was conducted at 37 °C for 30 min, in 15 μL of reaction buffer containing 20 mM HEPES-NaOH, pH 7.5,100 mM KCl, 2 mM MgCl_2_, 1 mM DTT, and 5% glycerol, and stopped by heating to 72 °C for 10 min. Cleavage products were resolved by electrophoresis on 1% agarose gel and visualized by GelDoc.

### In vivo cleavage assay

For in vivo plasmid cleavage, pCS27 plasmids containing SpCas9wt or SpNG-LWT were transferred into *E. coli* BW25113(F′) cells harboring the reporter plasmids. All transformants were plated on LB agar plates containing 50 mg/L kanamycin, 100 mg/L ampicillin, and 0.5 mM IPTG. For in vivo genome cleavage, pCS27 plasmids containing SpCas9wt or SpNG-LWT were transferred to *E. coli* BW25113(F′)::eGFP harboring pZE12-luc containing sgRNA targeting 5′-TGG-3′ (pZE-sgegfp-TGG) or 5′-CAT-3′ PAM (pZE-sgegfp-CAT). The transformants were plated on LB agar plates containing 50 mg/L kanamycin, 100 mg/L ampicillin, and 0.5 mM IPTG. As a control, equimolar pCS27 empty plasmid was transferred in both cleavage tests. The cleavage activity was calculated as $\left(1-\frac{{{colonies}\; {formed}\; {with}\; {Cas}}9}{{{colonies}\; {formed}\; {without}\; {Cas}}9}\right)$.

### HPLC analysis

Metabolites from shake flask cultivations were analyzed by Dionex Ultimate 3000 HPLC equipped with a Coregel-64H column (Transgenomic, Omaha, NE). One milliliter of sample from each shake flask culture was centrifuged at 21,130 × *g* for 10 min, and the supernatant was filtered through 0.22 μm membrane filter before HPLC analysis. The mobile solution was 4 mN sulfuric acid setting at a flow rate of 0.4 mL/min. The column oven temperature was set at 45 °C.

### MD simulation

To further compare SpCas9 or its variants accommodation with different PAM sequences, we conducted MD simulation by using GROMACS version 2018 and CHARMM36 force field^55^. First, the crystal structure of SpNG (PDB ID: 6AI6) was retrieved from RCSB’s Protein Data Bank (www.rcsb.org)^56^. The PAM sequence (5′-TGG-3′) of SpNG was virtually mutated into 5′-CAT-3′ to generate NG-CAT in software Maestro (Schrodinger, version 12.4). LWQT-NGG and LWQT-CAT were generated by mutating four amino acids (V1135L/S1136W/R1333Q/V1335T) in a PDB viewer. Then, we used CHARMM-GUI to build the MD simulation solution box, which was a cubic box with a length of 138 Å and filled with water molecules^57,58^. After energy minimization, the structures were equilibrated using an NVT ensemble (constant number of particles, volume, and temperature) and NPT ensemble (the number of particles, pressure, and temperature). The target equilibration temperature was 300 K. Finally, MD simulations were performed for 100 ns. After the MD simulations, we calculated the number of hydrogen bonds and the minimum distance between the native/mutated amino acids and nucleotides. The protein structures were visualized via PyMOL^59^.

### Statistics

No statistical method was used to predetermine sample size. All data for eGFP repression assay and shake flask experiments were presented as the mean ± standard deviation of biological triplicates (*n* = 3), which were also reported in the corresponding figure legends. The colonies used for data collection were randomly selected from the agar plates. For two-tailed *t*-test analysis of repression efficiency of SpdNG-LWQT and SpdRY, ten independent biological replicates (*n* = 10) were applied. Data were analyzed using Microsoft Excel and the two-tailed *t*-test was performed with JMP Pro 16 software. No data were excluded from the analyses and the investigators were not blinded to allocation during experiments and outcome assessment.

### Reporting summary

Further information on research design is available in the Nature Research Reporting Summary linked to this article.

## Supplementary information

Supplementary Information

Reporting Summary

## Data Availability

All data needed to evaluate the conclusions in the paper are present in the paper and/or the Supplementary Information. Plasmids from Addgene (#139498, #126717, and #60847) were used in this study. Structural information from PDB (ID: 6AI6 and 4UN3) was used in this study. The raw and/or processed data underlying the bar charts and uncropped gels generated in this study are provided in the Source Data file. Source Data are provided with this paper.

## Source data

Source Data
