# Supplementary material for: Engineering a PAM-flexible SpdCas9 variant as a universal gene repressor
Source: Nat Commun. 2021 Nov 25;12:6916. doi: 10.1038/s41467-021-27290-9 (PMC8617050; doi:10.1038/s41467-021-27290-9)
Supplement: Supplementary file 1 — Supplementary Information [file 41467_2021_27290_MOESM1_ESM.docx]

Supplementary Information for

**Engineering a PAM-flexible SpdCas9 variant as a universal gene repressor**

Wang *et al.*

**
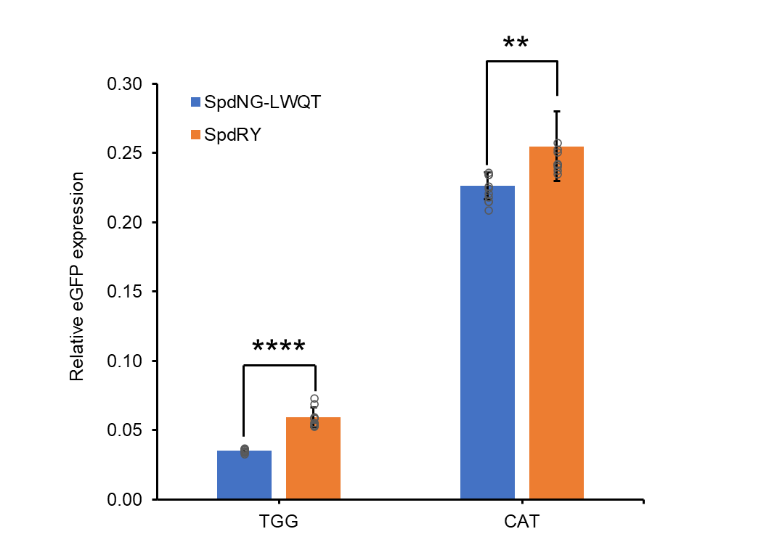
**

**Supplementary Figure 1. Comparison of eGFP repression activity of SpdNG-LWQT and SpdRY towards 5’-TGG-3’ and 5’-CAT-3’ PAMs.** *** P*≤ 0.01, *****P*≤ 0.0001 (two-tailed Student's *t*-test; *n* = 10 independent biological replicates).

**
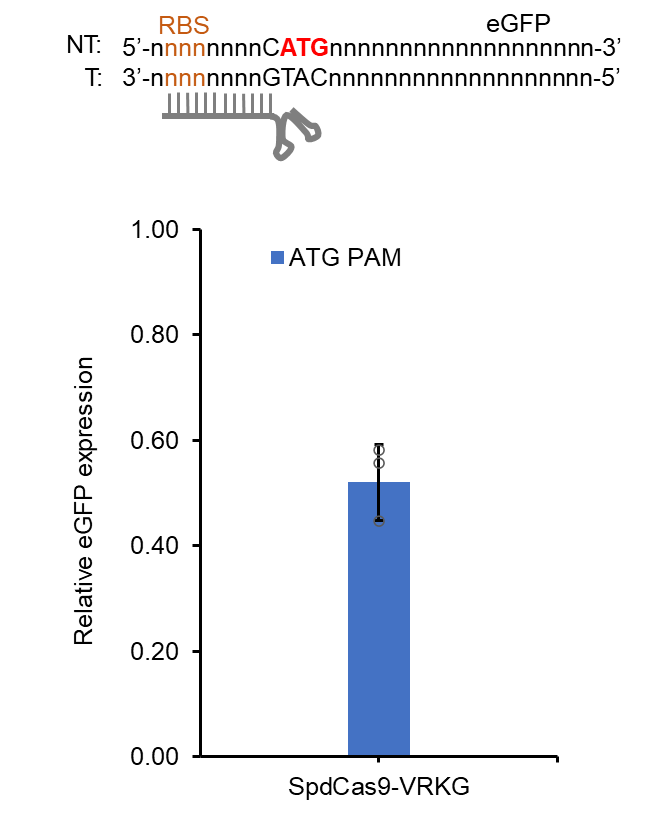
**

**Supplementary Figure 2. The eGFP repression assay with SpdCas9-VRKG targeting 5’-ATG-3’ PAM.** The PAM was ATG start codon on non-template DNA strand and the sgRNA was targeting template DNA strand. Data indicated the mean ± standard deviation (*n* = 3 independent biological replicates).

**Supplementary Table 1. Strains and plasmids used in this study.**

| **Strains** | **Description** | **Source** |
| --- | --- | --- |
| *E. coli* XL-1 Blue | recA1 endA1 gyrA96 thi-1 hsdR17 supE44 relA1 lac *F′* [*traD36* proAB lacI^q^Z∆M15 Tn10 (Tet^r^)] | Stratagene |
| *E. coli* BW25113(F′) | rrnBT14 ΔlacZWJ16 hsdR514 ΔaraBADAH33 *Δ*rhaBADLD78 *F′* [*traD36 proAB lacI^q^ZΔM15 Tn10(Tet^r^)*] | ^1^ |
| *E. coli* BW25113(F′)::eGFP | *E. coli* BW25113(F′) with *P_L_lacO1*-eGFP integrated in between the *nupG* and *speC* loci | ^2^ |
| *E. coli* BW25113(F′)::MVA | *E. coli* BW25113(F′) with *P_L_lacO1*-eGFP integrated at *aslB* locus | This study |
| *E. coli* BL21 Star(DE3) | F^-^ *ompT hsdSB (rB-mB-) gal dcm rne131* (DE3) | Invitrogen |
| *Saccharomyces cerevisiae* BY4741 | *MATa his3Δ1 leu2Δ0 met15Δ0 ura3Δ0* | ATCC |
| **Plasmids** | **Description** | **Reference** |
| pZE12-luc | *P_L_lacO1*, *colE ori*, *Amp^R^* | ^3^ |
| pCS27 | *P_L_lacO1*, *P15A ori*, *Kan^R^* | ^4^ |
| pSA74 | *P_L_lacO1*, *pSC101** ori, *Cl*^r^ | ^5^ |
| pETDuet-1 | T7, *pBR322 ori*, *Amp^R^* | Novagen |
| pSP571 | *2μ ori*, *HIS3* selection marker | Addgene |
| pZ_P-GAP-eGFP | Zeocin^R^ | Addgene |
| pCAS | *2μ ori*, *Kan^R^* | ^6^ |
| pCS-*Plpp1*-SpdCas9wt | pCS27 containing *Plpp1*-SpdCas9wt (D10A/H840A) | This study |
| pCS-*Plpp1*-dxCas9 3.7 | pCS27 containing *Plpp1*-SpdCas9 variant  A262T/R324L/S409I/E480K/E543D/M694I/E1219V | This study |
| pCS-*Plpp1*-SpdNG | pCS27 containing *Plpp1*-SpdCas9 variant  L1111R/D1135V/G1218R/E1219F/A1322R/R1335V/T1337R | This study |
| pCS-*Plpp1*-SpdRY | pCS27 containing *Plpp1*-SpdCas9 variant  A61R/G1104K/L1111R/D1135L/S1136W/G1218K/E1219Q/N1317R/A1322R/R1333P/R1335Q/T1337R | This study |
| pCS-*Plpp1*-SpdCas9-Q | pCS27 containing *Plpp1*-SpdCas9 variant R1333Q | This study |
| pCS-*Plpp1*-SpdCas9-VR | pCS27 containing *Plpp1*-SpdCas9 variant R1335V/T1337R | This study |
| pCS-*Plpp1*-SpdCas9-QVR | pCS27 containing *Plpp1*-SpdCas9 variant R1333Q/R1335V/T1337R | This study |
| pCS-*Plpp1*-SpdCas9-QTR | pCS27 containing *Plpp1*-SpdCas9 variant R1333Q/R1335T/T1337R | This study |
| pCS-*Plpp1*-dxCas9 3.7-Q | pCS27 containing *Plpp1*-dxCas9 3.7 variant  R1333Q | This study |
| pCS-*Plpp1*-dxCas9 3.7-VR | pCS27 containing *Plpp1*-dxCas9 3.7 variant  R1335V/T1337R | This study |
| pCS-*Plpp1*-dxCas9 3.7-QVR | pCS27 containing *Plpp1*-dxCas9 3.7 variant  R1333Q/R1335V/T1337R | This study |
| pCS-*Plpp1*-SpdNG-RT | pCS27 containing *Plpp1*-SpdNG variant  V1335R/R1337T | This study |
| pCS-*Plpp1*-SpdNG-QRT | pCS27 containing *Plpp1*-SpdNG variant  R1333Q/V1335R/R1337T | This study |
| pCS-*Plpp1*-SpdNG-Q | pCS27 containing *Plpp1*-SpdNG variant  R1333Q | This study |
| pCS-Plpp1-SpdNG-QG | pCS27 containing *Plpp1*-SpdNG variant  R1333Q/V1335G | This study |
| pCS-*Plpp1*-SpdNG-QA | pCS27 containing *Plpp1*-SpdNG variant  R1333Q/V1335A | This study |
| pCS-*Plpp1*-SpdNG-QS | pCS27 containing *Plpp1*-SpdNG variant  R1333Q/V1335S | This study |
| pCS-*Plpp1*-SpdNG-QT | pCS27 containing *Plpp1*-SpdNG variant  R1333Q/V1335T | This study |
| pCS-*Plpp1*-SpdNG-QL | pCS27 containing *Plpp1*-SpdNG variant  R1333Q/V1335L | This study |
| pCS-*Plpp1*-SpdNG-QI | pCS27 containing *Plpp1*-SpdNG variant  R1333Q/V1335I | This study |
| pCS-*Plpp1*-SpdNG-QV | pCS27 containing *Plpp1*-SpdNG variant  R1333Q/V1335V | This study |
| pCS-*Plpp1*-SpdNG-QM | pCS27 containing *Plpp1*-SpdNG variant  R1333Q/V1335M | This study |
| pCS-*Plpp1*-SpdNG-QC | pCS27 containing *Plpp1*-SpdNG variant  R1333Q/V1335C | This study |
| pCS-*Plpp1*-SpdNG-QF | pCS27 containing *Plpp1*-SpdNG variant  R1333Q/V1335F | This study |
| pCS-*Plpp1*-SpdNG-QY | pCS27 containing *Plpp1*-SpdNG variant  R1333Q/V1335Y | This study |
| pCS-*Plpp1*-SpdNG-QW | pCS27 containing *Plpp1*-SpdNG variant  R1333Q/V1335W | This study |
| pCS-*Plpp1*-SpdNG-QD | pCS27 containing *Plpp1*-SpdNG variant  R1333Q/V1335D | This study |
| pCS-*Plpp1*-SpdNG-QE | pCS27 containing *Plpp1*-SpdNG variant  R1333Q/V1335E | This study |
| pCS-*Plpp1*-SpdNG-QN | pCS27 containing *Plpp1*-SpdNG variant  R1333Q/V1335N | This study |
| pCS-*Plpp1*-SpdNG-QQ | pCS27 containing *Plpp1*-SpdNG variant  R1333Q/V1335Q | This study |
| pCS-*Plpp1*-SpdNG-QH | pCS27 containing *Plpp1*-SpdNG variant  R1333Q/V1335H | This study |
| pCS-*Plpp1*-SpdNG-QK | pCS27 containing *Plpp1*-SpdNG variant  R1333Q/V1335K | This study |
| pCS-*Plpp1*-SpdNG-KQT | pCS27 containing *Plpp1*-SpdNG variant  G1104K/R1333Q/V1335T | This study |
| pCS-*Plpp1*-SpdNG-TQT | pCS27 containing *Plpp1*-SpdNG variant  S1216T/R1333Q/V1335T | This study |
| pCS-*Plpp1*-SpdNG-VQT1 | pCS27 containing *Plpp1*-SpdNG variant  S1216V/R1333Q/V1335T | This study |
| pCS-*Plpp1*-SpdNG-VQT2 | pCS27 containing *Plpp1*-SpdNG variant  F1219V/R1333Q/V1335T | This study |
| pCS-*Plpp1*-SpdNG-AQT | pCS27 containing *Plpp1*-SpdNG variant  F1219A/R1333Q/V1335T | This study |
| pCS-*Plpp1*-SpdNG-QQT | pCS27 containing *Plpp1*-SpdNG variant  F1219Q/R1333Q/V1335T | This study |
| pCS-*Plpp1*-SpdNG-IQT | pCS27 containing *Plpp1*-SpdNG variant  V1135I/R1333Q/V1335T | This study |
| pCS-*Plpp1*-SpdNG-VWQT | pCS27 containing *Plpp1*-SpdNG variant  V1135V/S1136W/R1333Q/V1335T | This study |
| pCS-*Plpp1*-SpdNG-LWQT | pCS27 containing *Plpp1*-SpdNG variant  V1135L/S1136W/R1333Q/V1335T | This study |
| pCS-*Plpp1*-SpdNG-IWQT | pCS27 containing *Plpp1*-SpdNG variant  V1135I/S1136W/R1333Q/V1335T | This study |
| pCS-*Plpp1*-SpCas9wt | pCS27 containing *Plpp1*-SpCas9wt | This study |
| pCS-*Plpp1*-SpNG-LWQT | pCS27 containing *Plpp1*-SpNG variant  V1135L/S1136W/R1333Q/V1335T | This study |
| pCS-*Plpp1*-SpdNG-LWQTK | pCS27 containing *Plpp1*-SpdNG variant  V1135L/S1136W/R1333Q/V1335T/G1104K | This study |
| pCS-*Plpp1*-SpdCas9-VRKG | pCS27 containing *Plpp1*-SpdCas9 variant  D1135V/S1136R/D1332K/R1333G | This study |
| pETDuet-SpCas9wt | pETDuet-1 containing SpCas9wt | This study |
| pETDuet-SpNG-LWQT | pETDuet-1 containing SpNG-LWQT variant | This study |
| pCS-sgegfp-TGG | pCS27 containing sgegfp targeting non-template DNA strand towards 5’-TGG-3’ PAM contains spacer sequence: 5’- TGAGTAAAGGAGAAGAACTT-3’ | This study |
| pCS-sgegfp-CAT | pCS27 containing sgegfp targeting non-template DNA strand towards 5’-CAT-3’ PAM contains spacer sequence: 5’- TGAGTAAAGGAGAAGAACTT-3’ | This study |
| pZE-eGFP-sgegfp-TGG | pZE12-luc containing *P_L_lacO1*-egfp and *P_L_lacO1*-sgegfp-TGG in separate operons | This study |
| pZE-eGFP-sgegfp-CAT | pZE12-luc containing *P_L_lacO1*-egfp and *P_L_lacO1*-sgegfp-CAT in separate operons | This study |
| pZE-N-eGFP-sgegfp-CAT | pZE-eGFP-sgegfp-CAT with randomized N inserted before the start codon ATG of eGFP | This study |
| pZE-eGFP-sgegfp-CAT-TS | pZE12-luc containing *P_L_lacO1*-egfp and *P_L_lacO1*-sgegfp in separate operons, and sgegfp targeting template DNA strand towards 5’-CAT-3’ PAM contains spacer sequence: 5’- attaaagaggagaaaggtac-3’ | This study |
| pZE-eGFP-sgegfp-ATG-TS | pZE12-luc containing *P_L_lacO1*-egfp and *P_L_lacO1*-sgegfp in separate operons, and sgegfp targeting template DNA strand towards 5’-ATG-3’ PAM contains spacer sequence: 5’- ttaaagaggagaaaggtacc-3’ | This study |
| pZE-sgegfp-TGG | pZE12-luc containing sgegfp-TGG | This study |
| pZE-sgegfp-CAT | pZE12-luc containing sgegfp-CAT | This study |
| pSA-sgegfp-TGG | pSA74 containing sgegfp-TGG | This study |
| pSA-sgegfp-CAT | pSA74 containing sgegfp-CAT | This study |
| pCS-*Plpp1*-SpdNG-LWQT-sgegfp-TGG | pCS-*Plpp1*-SpdNG-LWQT containing sgegfp-TGG | This study |
| pCS-*Plpp1*-SpdRY-sgegfp-TGG | pCS-*Plpp1*-SpdRY containing sgegfp-TGG | This study |
| pCS-*Plpp1*-SpdNG-LWQT-sgegfp-CAT | pCS-*Plpp1*-SpdNG-LWQT containing sgegfp-CAT | This study |
| pCS-*Plpp1*-SpdRY-sgegfp-CAT | pCS-*Plpp1*-SpdRY containing sgegfp-CAT | This study |
| pZE-NNN-eGFP-sgegfp-CAT | pZE-eGFP-sgegfp-CAT with NNN inserted adjacent to the start codon ATG of eGFP | This study |
| pCS-*thl-mvaS-mvaA* | pCS27 containing *thl* from *Clostridium difficile*, *mvaS* from *Lactobacillus casei*, and *mvaA* from Ruegeria pomeroyi | This study |
| pZE-*sggltA*-TGG | pZE12-luc containing *sggltA* targeting 5’-TGG-3’ PAM | This study |
| pZE-*sgaccA*-TGG | pZE12-luc containing *sgaccA* targeting 5’-TGG-3’ PAM | This study |
| pZE-*sgfabD*-TGG | pZE12-luc containing *sgfabD* targeting 5’-TGG-3’ PAM | This study |
| pZE-*sggltA*-CAT | pZE12-luc containing *sggltA* targeting 5’-CAT-3’ PAM | This study |
| pZE-*sgaccA*-CAT | pZE12-luc containing *sgaccA* targeting 5’-CAT-3’ PAM | This study |
| pZE-*sgfabD*-CAT | pZE12-luc containing *sgfabD* targeting 5’-CAT-3’ PAM | This study |
| pSP571-yeGFP | pSP571 containing *P_GAP_-yeGFP* cassette amplified from pZ_P-GAP-eGFP | This study |
| pSP571-yeGFP-sgyegfp-TGG | pSP571-yeGFP containing sgRNA targeting yeGFP at 5’-TGG-3’ PAM | This study |
| pSP571-yeGFP-sgyegfp-CAT | pSP571-yeGFP containing sgRNA targeting yeGFP at 5’-CAT-3’ PAM | This study |
| pSP571-SpdCas9wt-yeGFP-sgyegfp-TGG | pSP571-yeGFP-sgyegfp-TGG containing *SpdCas9wt* under control of *P_GAP_* promoter | This study |
| pSP571-SpdCas9wt-yeGFP-sgyegfp-CAT | pSP571-yeGFP-sgyegfp-CAT containing *SpdCas9wt* under control of *P_GAP_* promoter | This study |
| pSP571-SpdRY-yeGFP-sgyegfp-TGG | pSP571-yeGFP-sgyegfp-TGG containing *SpdRY* under control of *P_GAP_* promoter | This study |
| pSP571-SpdRY-yeGFP-sgyegfp-CAT | pSP571-yeGFP-sgyegfp-CAT containing *SpdRY* under control of *P_GAP_* promoter | This study |
| pSP571-SpdNG-LWQT-yeGFP-sgyegfp-TGG | pSP571-yeGFP-sgyegfp-TGG containing *SpdNG-LWQT* under control of *P_GAP_* promoter | This study |
| pSP571-SpdNG-LWQT-yeGFP-sgyegfp-CAT | pSP571-yeGFP-sgyegfp-CAT containing *SpdNG-LWQT* under control of *P_GAP_* promoter | This study |

**References**

1. Atsumi, S. et al. Metabolic engineering of *Escherichia coli* for 1-butanol production. *Metab. Eng.* **10**, 305-311 (2008).

2. Yang, Y. et al. Sensor-regulator and RNAi based bifunctional dynamic control network for engineered microbial synthesis. *Nat. Commun.* **9**, 1-10 (2018).

3. Lutz, R. & Bujard, H. Independent and tight regulation of transcriptional units in *Escherichia coli* via the LacR/O, the TetR/O and AraC/I1-I2 regulatory elements. *Nucleic Acids Res.* **25**, 1203-1210 (1997).

4. Shen, C.R. & Liao, J.C. Metabolic engineering of *Escherichia coli* for 1-butanol and 1-propanol production via the keto-acid pathways. *Metab. Eng.* **10**, 312-320 (2008).

5. Huo, Y.-X. et al. Conversion of proteins into biofuels by engineering nitrogen flux. *Nat. Biotechnol.* **29**, 346-351 (2011).

6. Ryan, O.W. et al. Selection of chromosomal DNA libraries using a multiplex CRISPR system. *elife* **3**, e03703 (2014).
