# Supplementary material for: Engineering a PAM-flexible SpdCas9 variant as a universal gene repressor
Source: Nat Commun. 2021 Nov 25;12:6916. doi: 10.1038/s41467-021-27290-9 (PMC8617050; doi:10.1038/s41467-021-27290-9)
Supplement: Supplementary file 2 — Reporting Summary [file 41467_2021_27290_MOESM2_ESM.pdf]

nature research

Corresponding author(s): Yajun Yan

Last updated by author(s): 2021-11-8

Reporting Summary

Nature Research wishes to improve the reproducibility of the work that we publish. This form provides structure for consistency and transparency in reporting. For further information on Nature Research policies, see our Editorial Policies and the Editorial Policy Checklist.

Statistics

For all statistical analyses, confirm that the following items are present in the figure legend, table legend, main text, or Methods section.

n/a	Confirmed	
☐	☒	The exact sample size (*n*) for each experimental group/condition, given as a discrete number and unit of measurement
☐	☒	A statement on whether measurements were taken from distinct samples or whether the same sample was measured repeatedly
☐	☒	The statistical test(s) used AND whether they are one- or two-sided<br>*Only common tests should be described solely by name; describe more complex techniques in the Methods section.*
☒	☐	A description of all covariates tested
☒	☐	A description of any assumptions or corrections, such as tests of normality and adjustment for multiple comparisons
☐	☒	A full description of the statistical parameters including central tendency (e.g. means) or other basic estimates (e.g. regression coefficient) AND variation (e.g. standard deviation) or associated estimates of uncertainty (e.g. confidence intervals)
☐	☒	For null hypothesis testing, the test statistic (e.g. *F*, *t*, *r*) with confidence intervals, effect sizes, degrees of freedom and *P* value noted<br>*Give P values as exact values whenever suitable.*
☒	☐	For Bayesian analysis, information on the choice of priors and Markov chain Monte Carlo settings
☒	☐	For hierarchical and complex designs, identification of the appropriate level for tests and full reporting of outcomes
☒	☐	Estimates of effect sizes (e.g. Cohen's *d*, Pearson's *r*), indicating how they were calculated

Our web collection on statistics for biologists contains articles on many of the points above.

Software and code

Policy information about availability of computer code

Data collection	Data was collected using: Genesys 10S UV-Vis Spectrophotometer, NanoDrop 2000c spectrophotometer, Bio-Rad GelDoc system, Biotek Synergy microplate reader, and Dionex Ultimate 3000 HPLC.
Data analysis	Data was analyzed using Microsoft Excel Version 1902 (Build 11328.20420). The two-tailed t-test was performed with JMP Pro 16 software, GROMACS version 2018, Maestro software (Schrodinger, version 12.4), PyMOL software version 2.3.3.

For manuscripts utilizing custom algorithms or software that are central to the research but not yet described in published literature, software must be made available to editors and reviewers. We strongly encourage code deposition in a community repository (e.g. GitHub). See the Nature Research guidelines for submitting code & software for further information.

Data

Policy information about availability of data

All manuscripts must include a data availability statement. This statement should provide the following information, where applicable:

- Accession codes, unique identifiers, or web links for publicly available datasets
- A list of figures that have associated raw data
- A description of any restrictions on data availability

All data needed to evaluate the conclusions in the paper are present in the paper and/or the Supplementary Information. Plasmids from Addgene (#139498 [https://www.addgene.org/139498/], #126717 [https://www.addgene.org/126717/], and #60847 [https://www.addgene.org/60847/]) were used in this study. Structural information from PDB (ID: 6AI6 [https://www.rcsb.org/structure/6AI6], and 4UN3 [https://www.rcsb.org/structure/4UN3]) was used in this study. The raw and/or processed data underlying the bar charts and uncropped gels generated in this study are provided in the Source Data file. Source data are provided with this paper.

Field-specific reporting

Please select the one below that is the best fit for your research. If you are not sure, read the appropriate sections before making your selection.

- [x] Life sciences
- [ ] Behavioural & social sciences
- [ ] Ecological, evolutionary & environmental sciences

For a reference copy of the document with all sections, see nature.com/documents/nr-reporting-summary-flat.pdf

Life sciences study design

All studies must disclose on these points even when the disclosure is negative.

Sample size	No statistical methods were used to predict the sample size. Three biological replicates (n=3) were used for all cell-based assays (GFP fluorescence tests, in vivo plasmid cleavage efficiency assay in Fig. 3c and shake flask experiments). This size has previously been shown as sufficient to determine statistical differences in mean values of our investigated parameters (Nishimasu H, Shi X, Ishiguro S, et al. Engineered CRISPR-Cas9 nuclease with expanded targeting space[J]. Science, 2018, 361(6408): 1259-1262). For two-tailed t-test analysis of repression efficiency of SpdNG-LWQT and SpdRY (i.e. Supplementary Fig. 1), n=10 independent biological replicates were applied (A sufficiently large sample size (n≥10) has been previously used to compare the Cas9 efficiency: Behan F M, Iorio F, Picco G, et al. Prioritization of cancer therapeutic targets using CRISPR–Cas9 screens[J]. Nature, 2019, 568(7753): 511-516; Hackley C R. A Novel Set of Cas9 Fusion Proteins to stimulate Homologous Recombination: Cas9-HRs[J]. The CRISPR Journal, 2021, 4(2): 253-263.).
Data exclusions	No data was excluded from the statistical analysis.
Replication	All replication attempts in this study were successful. For each time, all cell-based assays (GFP fluorescence tests, in vivo plasmid cleavage efficiency assay in Fig. 3c and shake flask experiments) were performed in biological triplicates. For two-tailed t-test analysis of repression efficiency of SpdNG-LWQT and SpdRY (i.e. Fig. S1), n=10 independent biological replicates were applied. For in vitro DNA cleavage assay shown in Fig. 3b, in vivo genome cleavage assay in Fig. 3d, and CRISPRi assay on plates in Fig. 6b, two independent repeats were performed and both were successful.
Randomization	All bacterial cell cultures used for each experiments were grown under the same conditions, so randomization was not relevant.
Blinding	Cell cultures, sample processing and measurements were under the same conditions. As blinding was not relevant, investigators were not blinded to sample identity.

Reporting for specific materials, systems and methods

We require information from authors about some types of materials, experimental systems and methods used in many studies. Here, indicate whether each material, system or method listed is relevant to your study. If you are not sure if a list item applies to your research, read the appropriate section before selecting a response.

Materials & experimental systems

n/a	Involved in the study	
☒	☐	Antibodies
☒	☐	Eukaryotic cell lines
☒	☐	Palaeontology and archaeology
☒	☐	Animals and other organisms
☒	☐	Human research participants
☒	☐	Clinical data
☒	☐	Dual use research of concern

Methods

n/a	Involved in the study	
☒	☐	ChIP-seq
☒	☐	Flow cytometry
☒	☐	MRI-based neuroimaging
